# Effects of zinc deficiency and arsenic exposure on bone in growing female mice

**DOI:** 10.1038/s41598-026-72747-w

**Published:** 2026-09-27

**Authors:** Laura M. Beaver, Carmen P. Wong, Sarah Hasler, Maud Prati, Erica J. Dashner-Titus, Emily Ho, Adam J. Branscum, Russell T. Turner, Urszula T. Iwaniec

**Affiliations:** 1 Skeletal Biology Laboratory, School of Nutrition and Public Health, Oregon State University, Corvallis, OR USA; 2 Linus Pauling Institute, Oregon State University, Corvallis, OR USA; 3 Food Science and Technology, Oregon State University, Corvallis, OR USA; 4 Department of Pharmaceutical Sciences, College of Pharmacy, University of New Mexico, Albuquerque, NM USA

**Keywords:** Arsenite, Zinc, Arsenic, Mouse, Bone architecture, Microcomputed tomography, Diseases, Health care, Medical research

## Abstract

Arsenic contamination in groundwater often co-exists in regions prone to zinc deficiency. The goal of this research was to evaluate the independent and combined effects of zinc status [adequate, marginally-deficient, severely-deficient (30, 6, and < 0.5 mg Zn/kg diet)] and arsenic exposure (0, 50, or 500 ppb sodium arsenite) on bone in growing female mice (n = 7–8/treatment). Two experiments were conducted; the first focused on 4 weeks of severe zinc deficiency, the second on 6 weeks of marginal zinc deficiency. Bone response was evaluated in femurs using dual energy X-ray absorptiometry and microcomputed tomography. Severe zinc deficiency resulted in lower total femur bone mass, lower midshaft cortical thickness, and lower cancellous bone volume fraction in distal femur. Arsenite exposure did not significantly alter bone response to consuming a zinc-deficient diet. Neither marginal zinc deficiency nor combined marginal zinc deficiency and arsenite exposure affected the endpoints evaluated. In summary, severe but not marginal zinc deficiency, in growing mice, lowered bone mass and altered bone microarchitecture. In contrast, exposure to arsenite had little effect on bone in severely zinc-deficient, marginally zinc-deficient, or zinc-adequate mice.

**Supplementary Information:**

The online version contains supplementary material available at https://doi.org/10.1038/s41598-026-72747-w.

## Introduction

Zinc, a micronutrient which plays catalytic, structural and regulatory roles in protein function, is essential for optimal bone growth and maintenance^1–3^. Dietary zinc deficiency is common, estimated to occur in 17% of the global population, with incidence varying by geographical region^4^. Up to 30% of the population in South Asia is estimated to be zinc deficient^5^. The impact of zinc deficiency is particularly concerning in children as it is correlated with stunting (low height for age)^2,3,6^. Zinc deficiency can be effectively modeled using rodents and the adverse effects caused by zinc deficiency increase as the deficiency becomes more pronounced^7,8^. We recently established in rapidly growing rats that severe zinc deficiency, but not marginal zinc deficiency, impaired acquisition of cancellous and cortical bone, and caused abnormalities in bone microarchitecture^9^. Severe zinc deficiency in growing animals has also been shown to reduce total growth plate and hypertrophic zone thickness, linear bone growth, and bone strength^2^.

Zinc status and the health consequences of zinc deficiency can be influenced by co-exposure to environmental toxins, including inorganic arsenic. Arsenic is a metalloid that is widely distributed in the environment, including regions of the world where zinc deficiency can occur^10^. It is estimated that between 94 and 220 million people are exposed to high arsenic concentrations through household groundwater usage^11^. Regions with high arsenic levels in groundwater include Argentina, Bangladesh, Chile, China, India, Mexico, Vietnam and some regions in the United States, including indigenous lands^12,13^. War can also decrease access to zinc containing foods and be associated with arsenic exposure^14,15^. The World Health Organization has set a guideline for drinking water to contain no more than 10 μg/liter (10 ppb) arsenic but concentrations of 50 and 500 ppb have been found in wells^12,16^. Multiple forms of arsenic exist in nature and, among the inorganic forms, environmental exposure to arsenite is generally considered more toxic than exposure to similar concentrations of arsenate^17^.

Long-term exposure to inorganic arsenic in drinking water in children and adolescents is an important health concern and is associated with increased development of asthma, endothelial dysfunction, and cancer^18–22^. Less is known about how environmental exposure to inorganic arsenic affects skeletal health^23^. Arsenic accumulates in bone^24,25^ and individuals exposed to arsenic have been reported to have decreased bone mineral density^26–28^. However, individuals exposed to arsenic are often exposed to additional environmental contaminants like cadmium, thus complicating interpretation of results and the ability to establish causality to a specific toxic metal^28–34^. Other environmental factors, like nutritional deficiencies, may also impact the development of skeletal abnormalities in the presence of arsenic exposure, but this has not been fully explored^35^. This research is needed as co-exposure to zinc deficiency and arsenic in other tissues has been shown to increase oxidant stress, DNA double strand breaks, and inflammation, beyond what was observed with either condition alone^8,36–38^. There is evidence that inorganic arsenic exposure inhibits osteoblast differentiation, decreases endochondral ossification, and alters bone turnover in rodents, but findings are not consistent across studies^39–42^.

The goal of the current research was to assess the impact of zinc status and arsenite exposure on bone accrual. Specifically, we evaluated femur mass, geometry and cortical and cancellous bone microarchitecture in rapidly growing female mice. To accomplish our goal, we conducted 2 experiments. Experiment 1 focused on severe zinc deficiency and arsenic exposure. Experiment 2 focused on marginal zinc deficiency and arsenic exposure and utilized tissues from a previous study where zinc deficiency increased oxidative stress and arsenic exposure affected proinflammatory responses in the liver of the same animals^36^. As a control, we evaluated bone response to adequate zinc and arsenic exposure in both studies.

## Materials and methods

### Experimental design

All experimental protocols were approved by the Oregon State University Institutional Animal Care and Use Committee (IACUC). All methods were carried out in accordance with the IACUC approved protocols, the NIH Guide for the Care and the Use of Laboratory Animals and ARRIVE guidelines. Mice were housed in a room on standard light cycle (12 h light/dark cycle), standard housing temperature (22 °C), with water and food provided ad libitum. Mice in both experiments were acclimated for two days prior to initiation of the experiment. Sodium arsenite (NaAsO_2_, Sigma-Aldrich) was dissolved in deionized water (vehicle) and was provided in drinking water at the final concentrations of 0, 50 or 500 ppb of arsenite. These concentrations were chosen because arsenic can be found at 50–500 ppb concentrations in various ground water sources around the world and was shown to cause detectable levels of arsenic in tissues like skin^12,16,36^. Diets were based on a modified AIN-93G rodent diet formulated with egg white with zinc provided as zinc carbonate (Supplemental Table S1). These two models of zinc deficiency were explored because severe zinc deficiency is known to adversely affect the skeleton but is relatively rare while marginal zinc deficiency is common in human populations^43^.

### Experiment 1: Effects of severe zinc deficiency and arsenite exposure on cortical and cancellous bone microarchitecture in femur

Three-week-old female mice (C57BL/6J, The Jackson Laboratory) were acclimated for two days with ZA diet and then randomized into one of 6 groups (n = 8/group): (1) Zinc adequate (ZA; 30 mg Zn/kg diet) plus vehicle (deionized water, control animals), (2) ZA plus 50 ppb arsenite, (3) ZA plus 500 ppb arsenite, (4) severe zinc deficient (SZD; < 0.5 mg Zn/kg diet) plus vehicle, (5) SZD plus 50 ppb arsenite, or (6) SZD plus 500 ppb arsenite. One mouse in the SZD plus 500 ppb arsenite group died prior to completion of study and was not included in analysis. The mice were single housed in stainless steel suspended cages for the 4-week duration of treatment. Throughout the study body weights and dietary intakes of all mice were monitored.

### Experiment 2: Effects of marginal zinc deficiency and arsenite exposure on cortical and cancellous bone microarchitecture in femur

Experiment 2 evaluated archived femurs from a previously published study so the experimental protocol is described in detail elsewhere^36^. In brief, 4-week-old female mice (C57BL/6J, The Jackson Laboratory) were acclimated for two days with ZA diet and then randomized into one of 6 groups (n = 8/group): (1) Zinc adequate (ZA; 30 mg Zn/kg diet) plus vehicle (water, control animals), (2) ZA plus 50 ppb arsenite, (3) ZA plus 500 ppb arsenite, (4) marginal zinc deficient (MZD; 6 mg Zn/kg diet) plus vehicle, (5) MZD plus 50 ppb arsenite, or (6) MZD plus 500 ppb arsenite. The mice were single housed in polycarbonate cages for the 6-week duration of treatment.

The slight difference in starting age of animals between the two experiments (3 or 4 weeks at initiation) was based on availability of youngest weaned mice at time of order. For both experiments, the control diet was designed to contain 30 mg zinc/kg diet which is adequate for meeting zinc requirements in rodents^44–48^ (Research Diet Inc, New Brunswick NJ, USA, Supplemental Table S1)^49,50^. Zinc concentration in diets was confirmed by inductively coupled plasma optical emission spectrometry (ICP-OES).

### Sample collection

For tissue and plasma collection in both experiments, mice were euthanized by CO_2_ asphyxiation and samples collected between 8 a.m. and 12:00 p.m., with lights on at 7 a.m. Femurs were dissected out, cleaned of soft tissue, and placed in a solution of 70% ethanol for analysis using dual energy x-ray absorptiometry (DXA) and microcomputed tomography (µCT).

### Plasma zinc

Total zinc concentration in plasma was determined by ICP-OES as previously described^36^. Samples were digested overnight in 0.5 ml undiluted nitric acid (OmniTrace^®^, Millipore Sigma, Burlington, MA) and then diluted with Chelex-treated nanopure water (final concentration 10% (v/v) nitric acid). Samples were centrifuged and analyzed against known standards at the W.M. Keck Collaboratory for Plasma Spectrometry (Oregon State University, Corvallis, OR, USA) using the Prodigy High Dispersion ICP-OES instrument (Teledyne LABS, Mason, OH, USA). Mean levels for plasma zinc in normal mice (i.e., young mice consuming a zinc-adequate diet) range from 0.53 to 0.75 µg/ml^36,48,51^.

### Kidney arsenic

Arsenic content of kidneys was determined by inductively coupled plasma mass spectrometry (ICP-MS). Tissue samples were weighed and then digested overnight in 0.5 ml undiluted nitric acid (OmniTrace^®^ nitric acid, EMD Millipore, Billerica, MA) as previously described^36^. Digested samples were further diluted with Chelex-treated nanopure water to a final concentration of 10% (v/v) nitric acid and centrifuged to remove insoluble cellular materials. One ml of each sample digest was transferred to a microcentrifuge tube and sent for arsenic quantification using an Agilent ICP-QQQ instrument against known standards at the Arizona Laboratory for Emerging Contaminants (University of Arizona, Tucson, AZ, USA).

### Bone densitometry

Total femur bone mineral content (BMC, g), bone area (cm^2^), and bone mineral density (BMD, g/cm^2^) were measured using DXA and researchers were blinded to treatment group (Piximus 2, Lunar Corporation, Madison, WI, USA).

### Bone microcomputed tomography

**Fig. 1:**
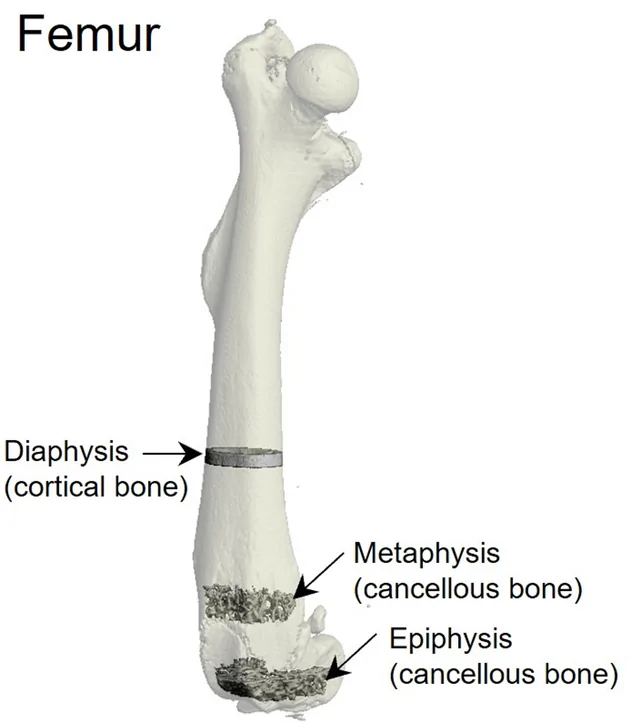
Regions of interest evaluated in the femur.

µCT was used for nondestructive three-dimensional evaluation of total bone volume and cancellous and cortical bone architecture. Researchers were blinded to treatment group and femurs were scanned in 70% ethanol using a Scanco µCT40 scanner (Scanco Medical AG, Bassendorf, Switzerland) at voxel size of 12 × 12 × 12 µm (55 k Vp x-ray voltage, 145 µA intensity, 200 ms integration time). Filtering parameters sigma and support were set 0.8 and 1, respectively. Bone segmentation was conducted at a threshold of 245 (scale, 0–1000) determined empirically. Total femur mineralized tissue volume (cortical + cancellous bone) was evaluated. This was followed by site-specific evaluation of cortical bone in the midshaft femur and cancellous bone in the distal femur metaphysis and epiphysis (Fig. 1). For the femoral diaphysis, 20 consecutive slices (240 µm) of bone distal to femur midshaft were evaluated. Cortical bone measurements included cross-sectional tissue volume (cortical and marrow volume, mm^3^), cortical volume (mm^3^), marrow volume (mm^3^), cortical thickness (µm), and polar moment of inertia (mm^4^, an index of bone strength in torsion). For the femoral metaphysis, 40 consecutive slices (480 µm) of cancellous bone, 45 slices (540 µm) proximal to growth plate/metaphysis boundary, were measured. The entire cancellous compartment was assessed in the femoral epiphysis. This consisted of 29 slices (± 1 slices) in the SZD experiment (Experiment 1) and 41 slices (± 0.4 slices) in the MZD experiment (Experiment 2). Irregular manual contouring a few pixels interior to the endocortical surface was used to delineate cancellous from cortical bone. Cancellous bone measurements in both the metaphysis and epiphysis included cancellous bone volume fraction (bone volume/tissue volume, %), connectivity density (mm^−3^), trabecular thickness (µm), trabecular number (mm^-1^), and trabecular spacing (µm).

### Statistical analysis

Dose–response data from the 2 × 3 factorial design relating zinc levels (zinc adequate versus moderate zinc deficient or severe zinc deficient) and arsenite levels (doses of 0, 50, or 500 ppb) were analyzed using two-way analysis of variance. Model diagnostics included use of the Breusch–Pagan test for homogeneity of variance, plots of residuals versus fitted values, normal quantile plots, and the Anderson–Darling test of normality. The Benjamini and Hochberg (1995) method for maintaining the false discovery rate at a maximum of 5% was used to adjust for multiple comparisons^52^. Data are presented as mean ± SE with individual mice represented by dots in figures. Differences were considered significant at p ≤ 0.05. Tendencies are reported at p > 0.05 but ≤ 0.10. Data analysis was performed using R version 4.12.

## Results

### Experiment 1: Effects of severe zinc deficiency and arsenite exposure on cortical and cancellous bone microarchitecture in femur

**Fig. 2:**
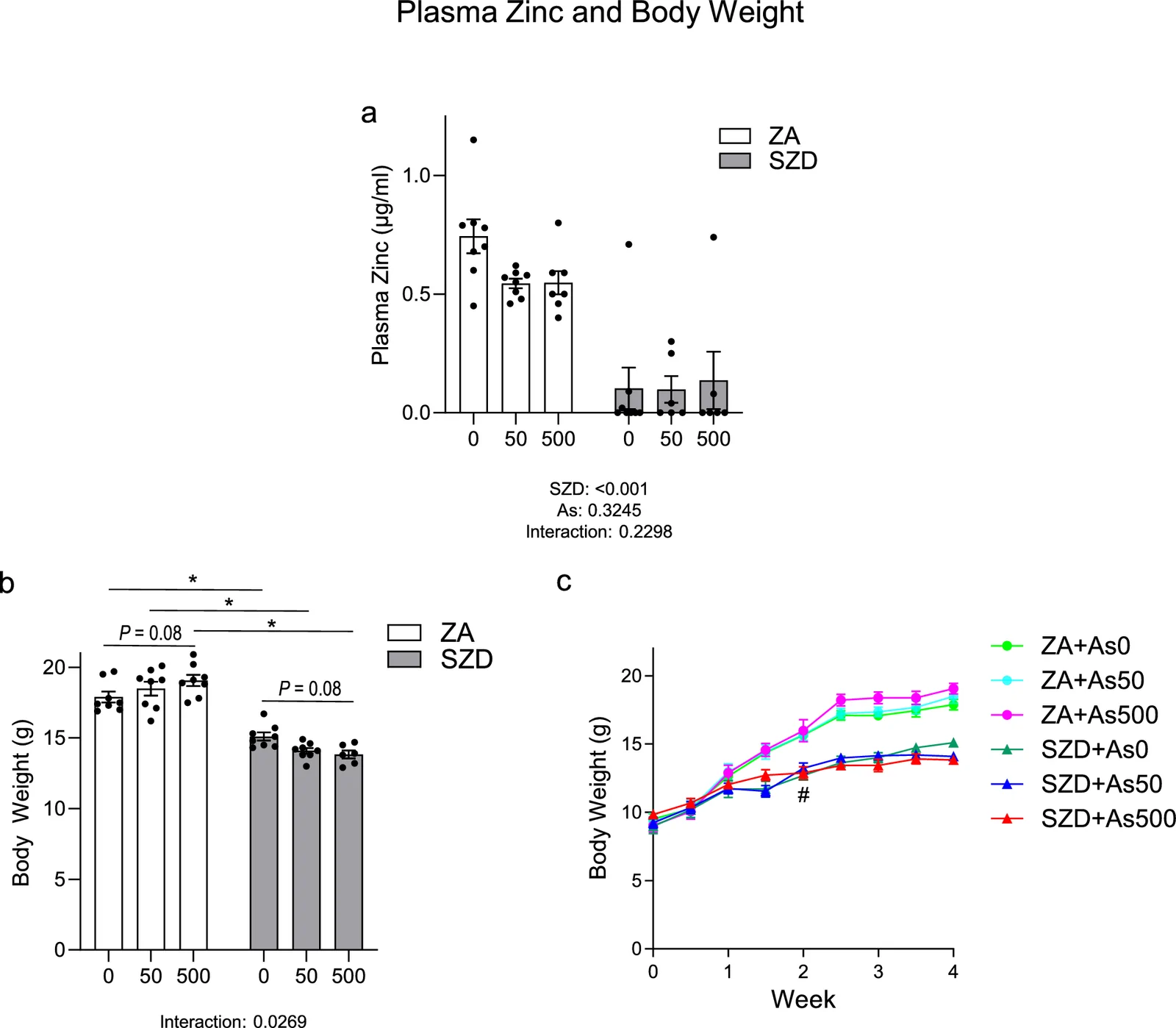
Severe zinc deficiency reduced plasma zinc concentrations and adversely affected body weight in growing female C57BL/6J mice. Effects of severe zinc deficiency and arsenite exposure (provided in drinking water at 0, 50, or 500 ppb) on plasma zinc concentrations (**a**) and body weight at termination of treatment (**b**) and longitudinally over the duration of treatment (**c**). Data are mean ± SE with individual data points shown as dots (**a,b**), n = 6–8/group. Reported *P*-values were calculated with a two-way analysis of variance and FDR-adjusted. Differences were considered significant at *P* ≤ 0.05. Tendencies are reported at *P* > 0.05 but ≤ 0.10. (**b**) * denotes a significant difference between specified treatment groups. (**c**) # indicates SZD + As 0 ppb, SZD + As 50 ppb, and SZD + As 500 ppb mice differed significantly from ZA mice at week 2 and all later timepoints. ZA, zinc adequate; As, arsenite, SZD, severe zinc deficient.

Plasma zinc: Plasma zinc status was assessed at completion of study. Plasma zinc levels were lower in SZD mice relative to ZA controls (Fig. 2a), confirming the SZD diet was effective in inducing zinc deficiency. Arsenite (As) exposure had no independent effect on plasma zinc, and there were no SZD x As interactions.

Body weight: The respective and combined effects of severe zinc deficiency and arsenite exposure on body weight are shown in Fig. 2b,c. A significant SZD x As interaction was noted for body weight at termination of treatment (Fig. 2b). Mice fed SZD diet had lower body weight compared to mice fed ZA diet with similar arsenite exposure. In addition, arsenite exposure at 500 ppb resulted in a tendency (*P* = 0.077) for higher body weight in ZA mice but lower body weight in SZD mice compared to their respective controls. A time course for changes in body weight with treatment can be appreciated in Fig. 2c. A divergence in body weight gain between ZA and SZD groups was apparent by 2 weeks of treatment. As expected, food intake was reduced in SZD groups (data not shown).

**Fig. 3:**
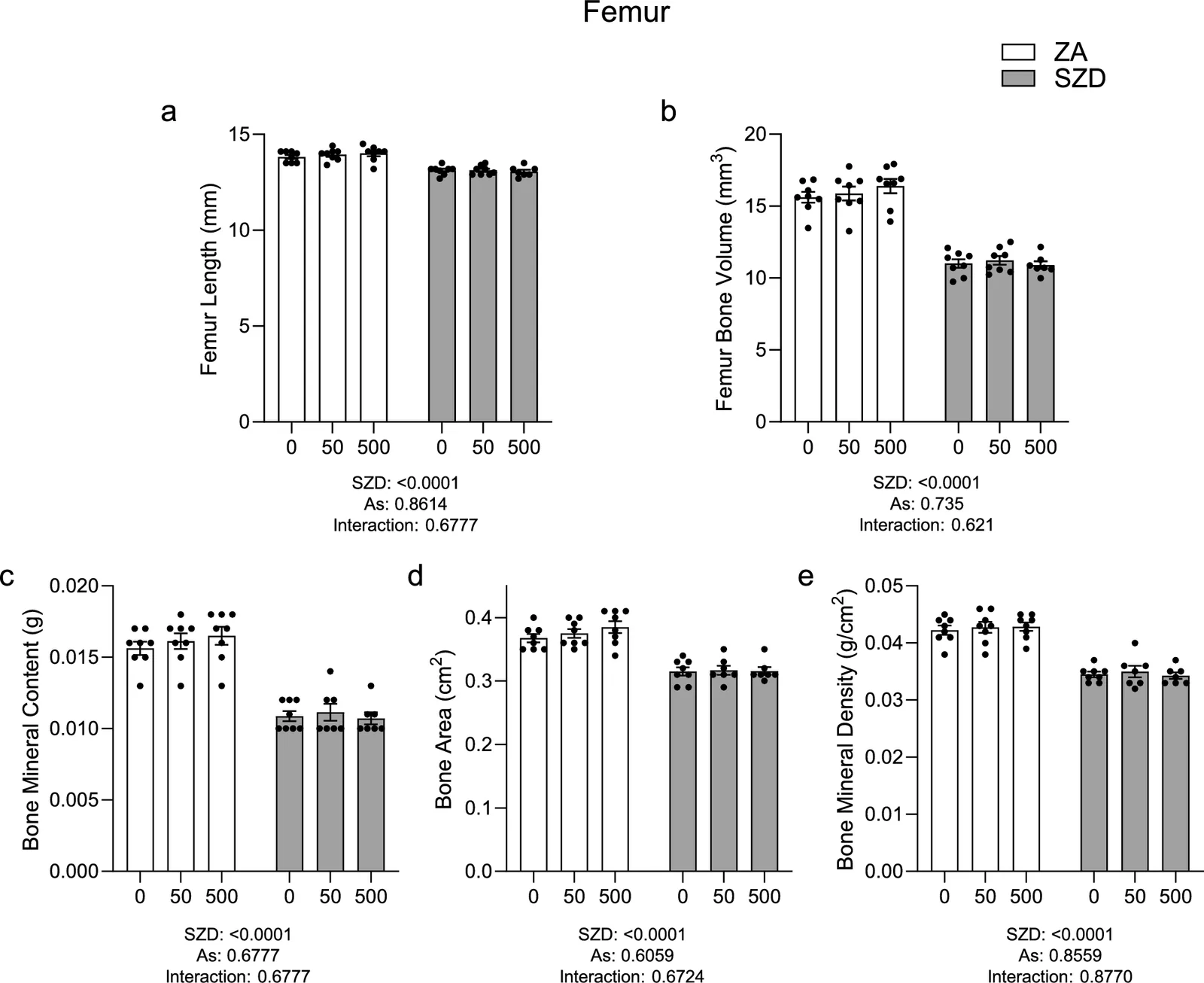
Severe zinc deficiency, but not arsenite exposure, adversely affected femur size and bone mass in growing female C57BL/6J mice. Effects of severe zinc deficiency and arsenite exposure (provided in drinking water at 0, 50, or 500 ppb) were quantified for femur length (**a**), total femur bone volume (**b**), total femur bone mineral content (**c**), bone area (**d**), and bone mineral density (**e**). Data are mean ± SE with individual data points shown as dots, n = 7–8/group. Reported *P*-values were calculated with a two-way analysis of variance and FDR-adjusted. Differences were considered significant at *P* ≤ 0.05. ZA, zinc adequate; As, arsenite, SZD, severe zinc deficient.

Femur: The respective and combined effects of severe zinc deficiency and arsenite exposure on femur length and femur bone volume, bone mineral content, bone area, and bone mineral density are shown in Fig. 3. SZD resulted in lower femur length (Fig. 3a), bone volume (Fig. 3b), bone mineral content (Fig. 3c), bone area (Fig. 3d), and bone mineral density (Fig. 3e). Arsenite exposure had no independent effect on any of the endpoints evaluated and there were no SZD x As interactions.

**Fig. 4:**
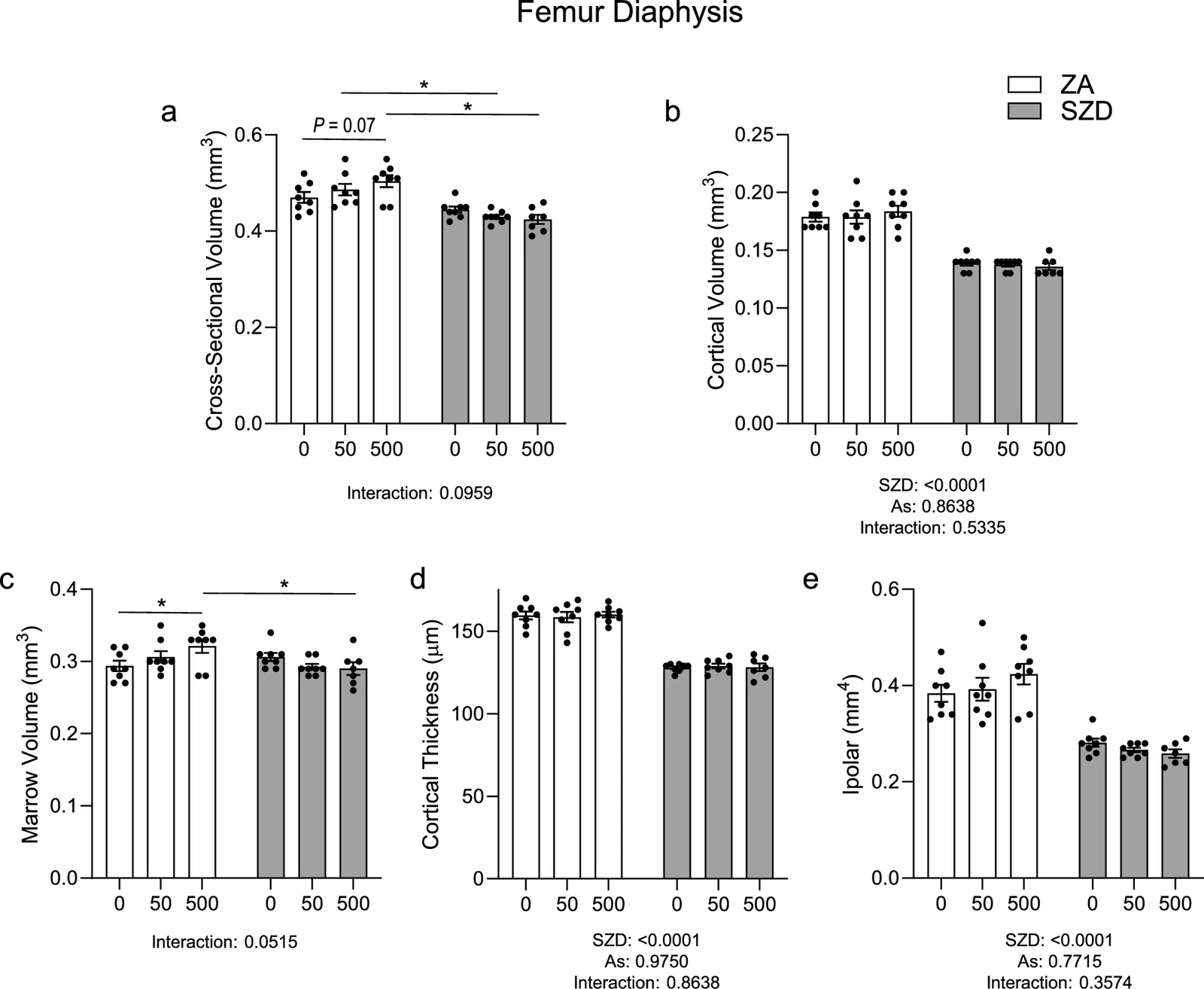
Severe zinc deficiency had a more robust effect than arsenite exposure on cortical bone microarchitecture in femur diaphysis in growing female C57BL/6J mice. Effects of severe zinc deficiency in combination with arsenite exposure (provided in drinking water at 0, 50, or 500 ppb) were quantified for cross-sectional volume (**a**), cortical bone volume (**b**), marrow volume (**c**), cortical thickness (**d**), and polar moment of inertia (**e**). Data are mean ± SE with individual data points shown as dots, n = 7–8/group. Reported *P*-values were calculated with a two-way analysis of variance and FDR-adjusted. Differences were considered significant at *P* ≤ 0.05. Tendencies are reported at *P* > 0.05 but ≤ 0.10. *Indicate significant difference between specified treatment groups. ZA, zinc adequate; As, arsenite exposure, SZD, severe zinc deficient.

Femur diaphysis: The respective and combined effects of severe zinc deficiency and arsenite exposure on cortical bone microarchitecture in the femur diaphysis are shown in Fig. 4. SZD resulted in lower cortical volume (Fig. 4b), cortical thickness (Fig. 4d), and polar moment of inertia (Fig. 4e). Arsenite exposure had no independent effect on any of the aforementioned endpoints and there were no significant SZD x As interactions. A tendency for an SZD x As interaction was noted for cross-sectional volume (*P* = 0.096) (Fig. 4a) and marrow volume (*P* = 0.052) (Fig. 4c). Cross-sectional volume was lower in SZD + 50 and SZD + 500 ppb As mice compared to their respective ZA controls. In addition, mice in the ZA + 500 ppb As group tended (*P* = 0.068) to have greater cross-sectional volume than ZA controls. Marrow volume was lower in SZD + 500 ppb As mice compared to ZA + 500 ppb As controls. In addition, mice in the ZA + 500 ppb As group had greater marrow volume than the ZA controls.

**Fig. 5:**
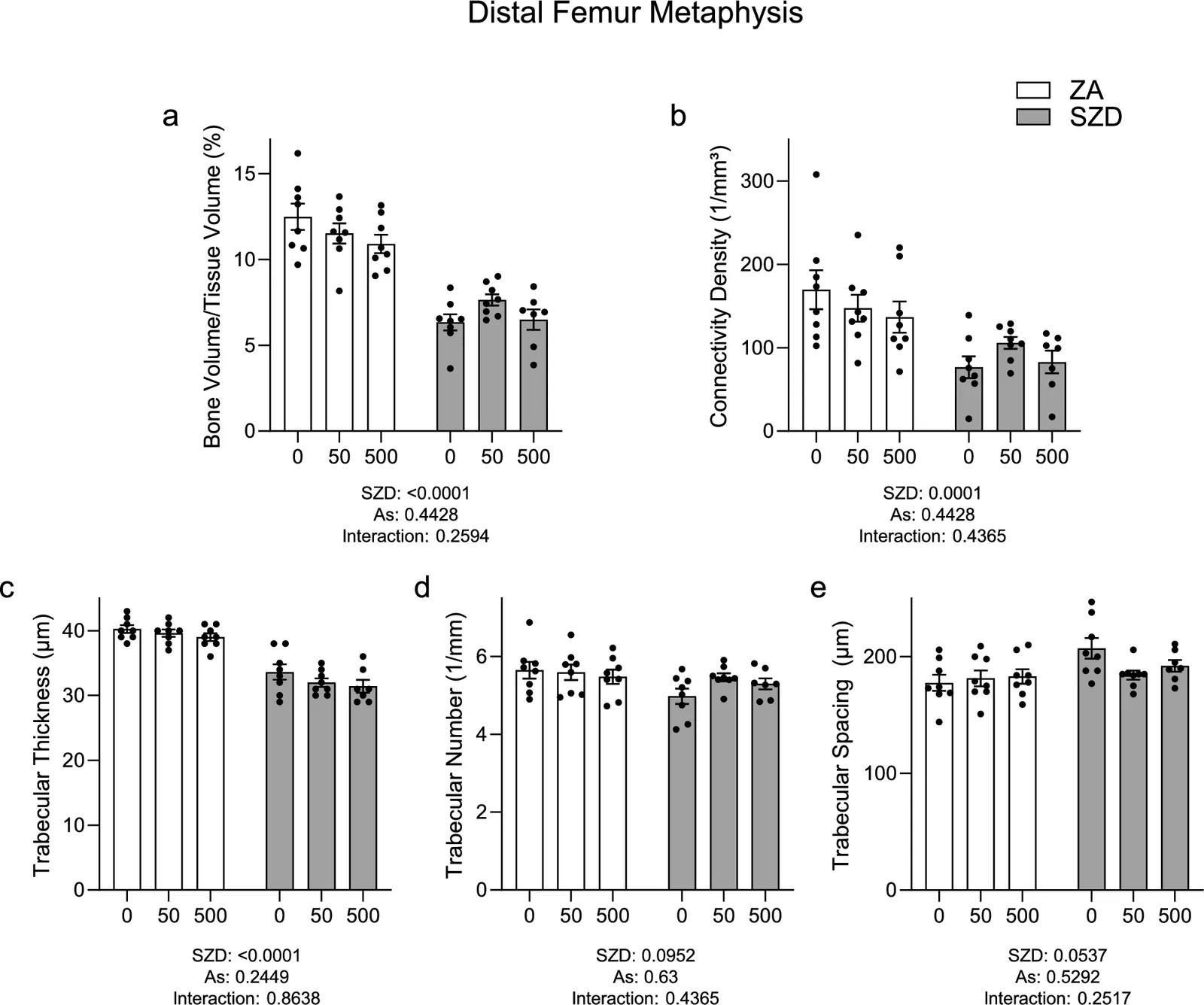
Severe zinc deficiency, but not arsenite exposure, adversely effected cancellous bone microarchitecture in distal femur metaphysis of growing female C57BL/6J mice. Effects of severe zinc deficiency in combination with arsenite exposure (provided in drinking water at 0, 50, or 500 ppb) were quantified for cancellous bone volume fraction (**a**), connectivity density (**b**), trabecular thickness (**c**), trabecular number (**d**), and trabecular spacing (**e**). Data are mean ± SE with individual data points shown as dots, n = 7–8 / group. Reported *P*-values were calculated with a two-way analysis of variance and FDR-adjusted. Differences were considered significant at *P* ≤ 0.05. Tendencies are reported at *P* > 0.05 but ≤ 0.10. ZA, zinc adequate; As, arsenite, SZD, severe zinc deficient.

Femur metaphysis: The respective and combined effects of severe zinc deficiency and arsenite exposure on cancellous bone microarchitecture in the distal femur metaphysis are shown in Fig. 5. SZD resulted in lower cancellous bone volume fraction (Fig. 5a), connectivity density (Fig. 5b), and trabecular thickness (Fig. 5c), and a tendency for lower trabecular number (*P* = 0.095, Fig. 5d) and higher trabecular spacing (*P* = 0.054, Fig. 5e). Arsenite exposure had no independent effect on any of the metaphyseal endpoints evaluated, and there were no SZD x As interactions.

**Fig. 6:**
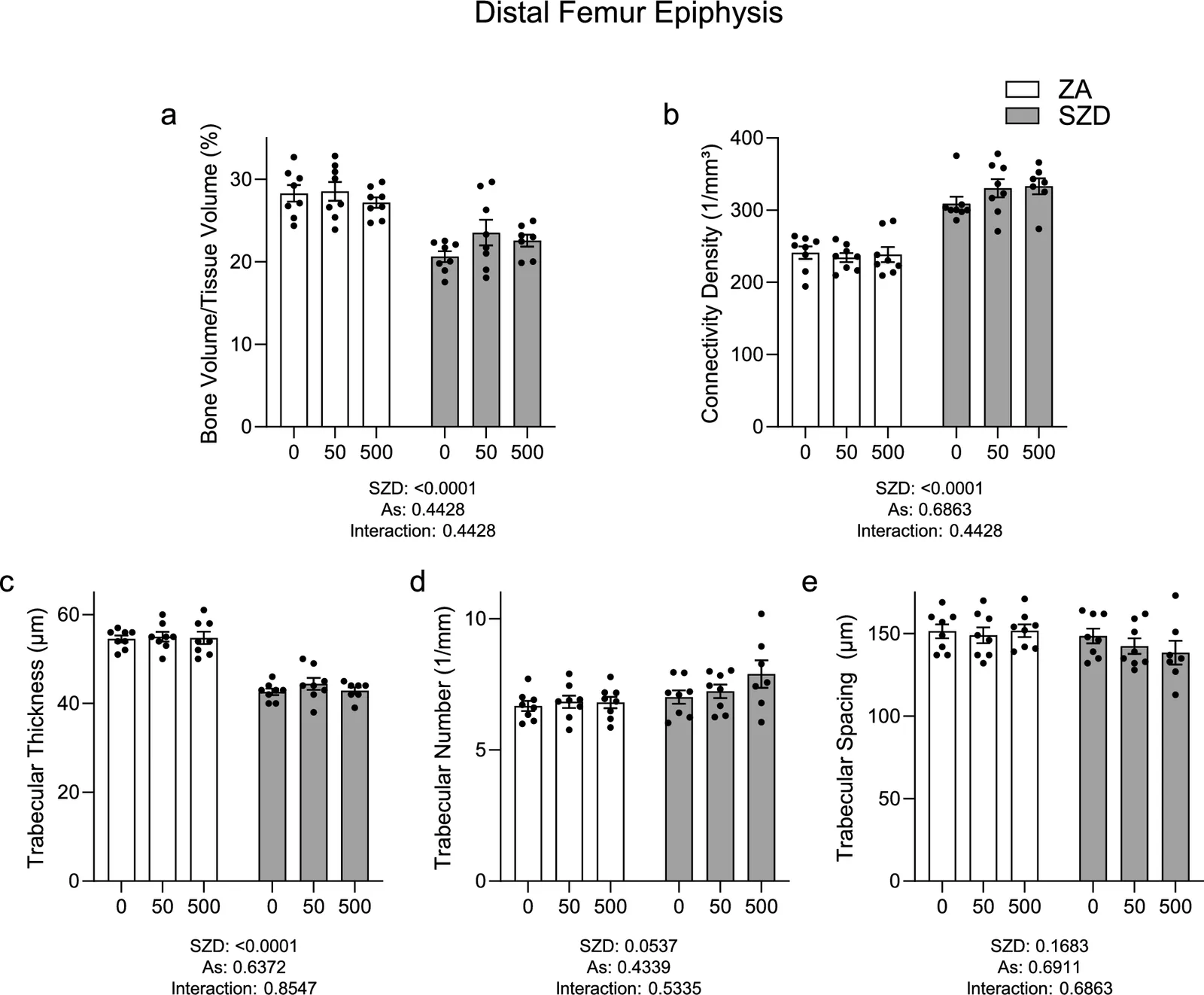
Severe zinc deficiency, but not arsenite exposure, adversely effected cancellous bone microarchitecture in distal femur epiphysis of growing female C57BL/6J mice. Effects of severe zinc deficiency in combination with arsenite exposure (provided in drinking water at 0, 50, or 500ppb) were quantified for cancellous bone volume fraction (**a**), connectivity density (**b**), trabecular thickness (**c**), trabecular number (**d**), and trabecular spacing (**e**). Data are mean ± SE with individual data points shown as dots, n = 7–8/group. Reported *P*-values were calculated with a two-way analysis of variance and FDR-adjusted. Differences were considered significant at *P* ≤ 0.05. Tendencies are reported at *P* > 0.05 but ≤ 0.10. ZA, zinc adequate; As, arsenite, SZD, severe zinc deficient.

Femur epiphysis: The respective and combined effects of severe zinc deficiency and arsenite exposure on cancellous bone microarchitecture in the distal femur epiphysis are shown in Fig. 6. SZD resulted in lower cancellous bone volume fraction (Fig. 6a), greater connectivity density (Fig. 6b), and lower trabecular thickness (Fig. 6c), and a tendency (*P* = 0.054) for greater trabecular number (Fig. 6d). Significant differences in response to treatment were not observed for trabecular spacing (Fig. 6e). Arsenite exposure had no independent effect on any of the epiphyseal endpoints evaluated, and there were no SZD x As interactions.

### Experiment 2: Effects of marginal zinc deficiency and arsenite exposure on cortical and cancellous bone microarchitecture in femur

**Fig. 7:**
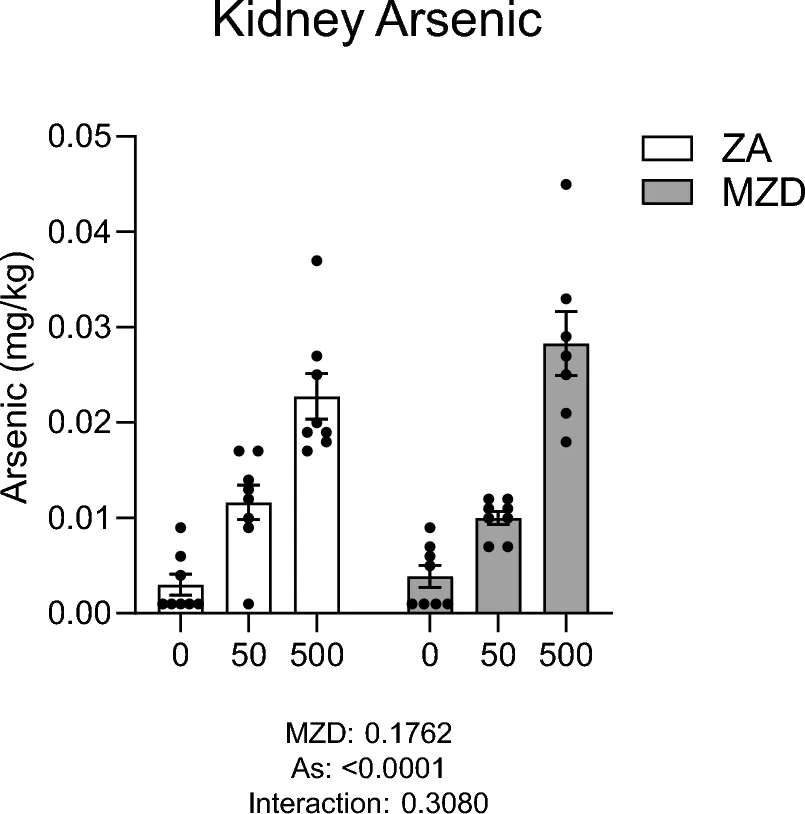
Arsenite exposures increased concentrations of arsenic in the kidneys of growing female C57BL/6J mice. Effects of marginal zinc deficiency in combination with arsenite exposure (provided in drinking water at 0, 50, or 500 ppb) were quantified for kidney using ICP-MS (mg of arsenic/kg kidney tissue). Data are mean ± SE with individual data points shown as dots, n = 7–8/group. Reported *P*-values were calculated with a two-way analysis of variance and FDR-adjusted. Differences were considered significant at *P* ≤ 0.05. As, arsenite; MZD, marginal zinc deficient; ZA, zinc adequate.

We next assessed the effect of 6 weeks of marginal zinc deficiency, with and without arsenite exposure, on bone status in growing female mice. Exposure to 50 and 500 ppb dose of arsenite increased arsenic concentrations in the kidney (Fig. 7). Plasma zinc status in response to treatment in these animals has been reported elsewhere^36^ and is shown in Table 1. Consumption of the MZD diet caused changes in expression of zinc transporters^51^, a modest but significant decrease in plasma zinc levels, and arsenite exposure resulted in further reduction in plasma zinc levels^36^.

**Table 1:** Effects of marginal zinc deficiency (MZD) in combination with arsenite (As) exposure (provided in the drinking water at 0, 50, or 500 ppb) on body weight, femur mass, and cortical bone architecture in femur diaphysis and cancellous bone architecture in distal femur metaphysis and epiphysis.

Treatment	ZA + 0 ppb As^1,2^	ZA + 50 ppb As	ZA + 500 ppb As	MZD + 0 ppb As	MZD + 50 ppb As	MZD + 500 ppb As	Zinc status P value	Arsenite P value	Interaction P value
Plasma									
Zinc (µg/mL)^3^	0.60 ± 0.08	0.47 ± 0.04	0.50 ± 0.03	0.52 ± 0.07	0.39 ± 0.03	0.45 ± 0.04	0.05	0.05	0.94
Body weight									
Weight (g)	18.2 ± 0.3	19.4 ± 0.6	18.7 ± 0.9	18.7 ± 0.4	19.3 ± 0.6	18.9 ± 0.6	0.96	0.84	0.96
Densitometry									
Ex vivo total femur									
Bone area (cm^2^)	0.38 ± 0.01	0.39 ± 0.01	0.38 ± 0.01	0.39 ± 0.01	0.40 ± 0.01	0.39 ± 0.01	0.80	0.80	0.99
Bone mineral content (g)	0.016 ± 0.001	0.017 ± 0.001	0.016 ± 0.001	0.016 ± 0.001	0.017 ± 0.001	0.017 ± 0.001	0.80	0.80	0.99
Bone mineral density (g/cm^2^)	0.041 ± 0.001	0.043 ± 0.001	0.042 ± 0.001	0.042 ± 0.001	0.043 ± 0.001	0.042 ± 0.001	0.80	0.80	0.96
microComputed Tomography									
Total femur									
Length (mm)	14.1 ± 0.2	14.2 ± 0.2	14.0 ± 0.2	14.1 ± 0.1	14.4 ± 0.1	14.3 ± 0.1	0.80	0.93	0.85
Bone volume (mm^3^)	15.1 ± 0.5	16.0 ± 0.5	15.6 ± 0.4	15.5 ± 0.5	16.5 ± 0.5	16.1 ± 0.7	0.80	0.80	0.99
Midshaft femur (cortical bone)									
Cross-sectional volume (mm^3^)	0.43 ± 0.01	0.45 ± 0.01	0.44 ± 0.01	0.44 ± 0.01	0.45 ± 0.01	0.45 ± 0.01	0.80	0.80	0.99
Cortical volume (mm^3^)	0.16 ± 0.00	0.17 ± 0.01	0.17 ± 0.00	0.17 ± 0.00	0.17 ± 0.00	0.18 ± 0.01	0.84	0.80	0.99
Marrow volume (mm^3^)	0.26 ± 0.01	0.27 ± 0.01	0.27 ± 0.02	0.27 ± 0.00	0.28 ± 0.00	0.27 ± 0.01	0.84	0.80	0.96
Cortical thickness (µm)	156 ± 4	159 ± 5	160 ± 3	156 ± 3	161 ± 4	163 ± 5	0.96	0.84	0.99
I_polar_ (mm^4^)	0.32 ± 0.01	0.35 ± 0.02	0.34 ± 0.01	0.34 ± 0.01	0.33 ± 0.01	0.36 ± 0.03	0.96	0.88	0.80
Distal femur metaphysis (cancellous bone)									
Bone volume/Tissue volume (%)	6.4 ± 0.8	6.3 ± 0.6	7.6 ± 1.1	6.2 ± 0.8	6.2 ± 0.7	5.9 ± 0.6	0.80	0.96	0.93
Connectivity density (1/mm^3^)	50 ± 15	51 ± 13	81 ± 22	46 ± 12	39 ± 11	46 ± 11	0.80	0.84	0.93
Trabecular thickness (µm)	38 ± 1	40 ± 2	39 ± 1	39 ± 1	42 ± 2	39 ± 1	0.93	0.80	0.96
Trabecular number (1/mm)	4.6 ± 0.1	4.5 ± 0.2	4.7 ± 0.3	4.6 ± 0.1	4.4 ± 0.2	4.4 ± 0.1	0.80	0.96	0.96
Trabecular spacing (µm)	221 ± 7	225 ± 10	217 ± 13	220 ± 6	230 ± 10	231 ± 8	0.84	0.96	0.96
Distal femur epiphysis (cancellous bone)									
Bone volume/Tissue volume (%)	26.6 ± 0.9	27.7 ± 0.6	27.6 ± 0.8	27.6 ± 0.8	27.2 ± 0.6	26.3 ± 0.7	0.96	0.96	0.80
Connectivity density (1/mm^3^)	205 ± 16	203 ± 13	216 ± 11	215 ± 10	173 ± 13	181 ± 17	0.80	0.80	0.80
Trabecular thickness (µm)	55 ± 1	57 ± 1	55 ± 0	56 ± 1	60 ± 1	58 ± 2	0.80	0.80	0.96
Trabecular number (1/mm)	5.6 ± 0.2	5.6 ± 0.2	5.7 ± 0.2	5.6 ± 0.2	5.4 ± 0.2	5.5 ± 0.2	0.93	0.96	0.96
Trabecular spacing (µm)	177 ± 5	175 ± 6	171 ± 5	170 ± 5	180 ± 5	179 ± 6	0.96	0.96	0.84

^1^Data are mean ± SE, n = 7–8/group.

^2^Marginal zinc deficiency (MZD), Zinc Adequate (ZA).

^3^Data previously published in graphic form^36^.

The respective and combined effects of marginal zinc deficiency and arsenite exposure on body weight, femur mass and length, cortical bone microarchitecture in femur diaphysis, and cancellous bone microarchitecture in distal femur metaphysis and epiphysis are shown in Table 1. Neither marginal zinc deficiency nor arsenite exposure had an independent effect on body weight, femur area, bone mineral content, bone mineral density, bone volume, or length and there were no MZD x As interactions. Likewise, neither marginal zinc deficiency nor arsenite exposure had an independent effect on any of the cortical endpoints evaluated in the femur diaphysis (cross-sectional volume, cortical volume, marrow volume, cortical thickness, and polar moment of inertia) or cancellous endpoints (cancellous bone volume fraction, connectivity density, trabecular thickness, trabecular number, and trabecular spacing) evaluated in the distal femur metaphysis and epiphysis. In addition, there were no MZD x As interactions for any of the cortical and cancellous bone endpoints assessed.

## Discussion

This study evaluated the effects of zinc status and arsenic exposure on bone mass and cortical and cancellous bone microarchitecture in femur in rapidly growing female mice. Four weeks of severe, but not 6 weeks of marginal zinc deficiency, lowered weight gain and resulted in a deficit in bone mass in femur, which was associated with altered cortical and cancellous bone microarchitecture. The inhibitory effect of severe zinc deficiency on weight gain was evident within 2 weeks of treatment. At termination of treatment, femur length, mass, and density were lower in mice fed severely zinc-deficient diet compared to mice fed zinc adequate diet. Architectural changes in femur of the severely zinc-deficient mice included lower cortical bone volume, thickness, and polar moment of inertia in femur diaphysis and lower cancellous bone volume fraction and trabecular thickness in distal femur metaphysis and epiphysis. In contrast to the skeletal effects of severe zinc deficiency, exposure of mice to arsenite at 50 or 500 ppb for either 4 or 6 weeks had minimal effect on bone mass and microarchitecture, irrespective of zinc status (adequate, marginally deficient or severely deficient).

Dietary zinc is essential for skeletal growth and maintenance of bone homeostasis^2,3,53,54^. The responses to zinc deficiency observed in the present study in growing female mice are very similar to those observed in growing male rats where severe but not marginal zinc deficiency suppressed body weight gain and linear and radial bone growth^9^. We did not assay biochemical markers of bone turnover in the mice in the current study but serum osteocalcin and C-terminal telopeptide of type I collagen, markers of bone formation and resorption, respectively, were reduced in severely zinc-deficient rats^9^. This earlier finding suggests that severe zinc deficiency suppresses bone resorption as well as bone formation. If this interpretation is correct, the resulting bone deficits observed in the present study are likely due to reduced bone accrual and not increased bone resorption.

The mechanisms by which severe zinc deficiency negatively affects the skeleton are likely to be both direct (e.g., reduced zinc in skeletal tissue) and indirect (e.g., reduced food consumption)^9,44,45^. Zinc repletion, following a period of severe zinc depletion, can be achieved by dietary zinc supplementation. In growing rats, zinc repletion caused accelerated weight gain, compared to zinc adequate controls, but failed to restore normal bone mass^9,44^. This suggests that neither zinc repletion nor weight gain is sufficient to fully counteract the detrimental effects of prior severe zinc deficiency on bone. This finding emphasizes the importance of maintaining adequate zinc status during skeletal growth.

Little is known regarding how environmental toxins such as arsenic influence the adverse skeletal effects of inadequate zinc intake. This is an important gap in knowledge as many of the mechanisms by which arsenic is known to cause damage are through cellular pathways that are also adversly affected by zinc deficiency, including inducing reactive oxygen species production and disrupting DNA repair^8^. Additionally arsenic has been shown to displace zinc from enzymes, affecting enzymatic function ^55–59^. The combination of zinc status and arsenic exposure has been shown to adversly affect zinc concentrations, and increase oxidant stress, DNA double strand breaks, and inflammation, and alter microbiome diversity beyond what was observed with either condition alone^8,36–38,51,60^, suggesting a combined exposure could also have been detrimental for the skeleton.

Here we investigated the independent and combined effects of 2 environmentally relevant exposures of arsenic in drinking water and 3 levels of dietary zinc on skeletal growth. The arsenite doses used in the present study resulted in detectable levels of arsenic in the kidney, and were reported to have biological effects, including decreasing plasma zinc levels and increasing an acute proinflammatory response in liver cells^36^. However, in contrast to the detrimental skeletal effects of severe zinc deficiency, arsenic exposure had no independent effect on femur mass, density or cancellous bone microarchitecture after 4 or 6 weeks of treatment. Increases in femur cross-sectional (*P* = 0.07) and marrow volume (*P* = 0.04) in mice fed adequate zinc and 500 ppb arsenic observed after 4 weeks of treatment were not detected after 6 weeks of treatment. Furthermore, other key indices of cortical bone strength, including cortical volume, cortical thickness, or polar moment of inertia did not change with either 4 or 6 weeks of treatment. Taken together, no compelling evidence was found for a negative effect of arsenite exposure on bone growth in female mice at the doses, exposure lengths, and treatments tested.

Adults who suffered from accidental arsenic poisoning (contaminated milk powder) as infants had decreased stature^61^. Severe arsenic poisoning in a patient was also shown to result in bone marrow toxicity^25^. While it is clear that these acute exposures to arsenic can be detrimental for skeletal health, the data are less consistent among studies evaluating subchronic and chronic arsenic exposures. For example, a cross-sectional study found no association between arsenic exposure and BMD in the general population^30^. Arsenic exposure in men (through work involving arsenic production or use) was shown in another study to be associated with an increased risk to develop osteopenia at an early age but an association between arsenic levels (hair) and BMD was not detected^26^. Subgroup analysis comparing arsenic levels in individuals with lower or higher BMD led to mixed results^26^. In a study using machine learning, arsenic levels in hair had a minor impact on model performance for predicting osteoporosis^27^. In another study using machine learning, urinary arsenic was negatively associated with BMD^28^. However, the higher and lower BMD study populations were poorly matched for age and BMI. Taken together, acute exposures to high levels of arsenic are detrimental to bone health but it is unclear what is the minimum concentration and duration of inorganic arsenite exposure in drinking water that would cause adverse skeletal health. Furthermore, people are exposed to additional environmental contaminants like cadmium which could act independently or in concert with arsenic to influence bone metabolism^17,23^.

Animal studies have been used to investigate actions of arsenic on bone. Aybar et al., reported that 45 days of arsenite in drinking water (10,000 ppb or 0.21 mg/kg body weight/day) had no effect on cancellous bone volume fraction but increased growth plate thickness and altered hematological parameters in blood^41^. While we did not measure white blood cells or growth plate thickness, we found no effect on femur length but similarly report no effect of arsenite (50 and 500 ppb) on cancellous bone architecture. Taken together, these findings suggest that subchronic exposure to arsenite, over a wide dose range, has no negative effect on cancellous architecture in long bones. It is possible that zinc status influences the skeletal response to arsenite. However, we found no compelling evidence to support this possibility. Arsenic exists in many chemical combinations with different levels of toxicity. The present study modeled arsenite exposure in drinking water. However, it is possible the chemical form of arsenic or mode of exposure (oral, dermal, or lung) influence the skeletal response. In support, intraperitoneal delivery (4 weeks) of high doses (5 and 10 mg/kg) of arsenic trioxide to rats to model chemotherapy exposure had detrimental effects on bone.^42^ Administration of arsenic trioxide for 12 weeks in drinking water at lower doses (50 and 500 ppb) to model environmental exposure also had detrimental effects on bone.^40^.

Our studies were limited to rapidly growing female mice exposed to a single form of inorganic arsenic for 4 or 6 weeks at two concentrations. Additional studies will be required to understand how the skeletal effects of zinc status and arsenic exposure may be influenced by species, sex, life stage (e.g., growing, adult, aged), strain, duration, or route of exposure. As mentioned, there is evidence that the form of arsenic, duration of exposure, and/or concentration and manner of exposure influences skeletal response^42^. The present study evaluated zinc status and arsenite exposure. However, co-exposure to multiple environmental toxins is common and dietary habits may also affect outcomes. For example, zinc deficiency, has also been shown to increase accumulation of lead in the calvarium and femur of growing rats^62–64^, while zinc supplementation has been shown to protect bone from cadmium exposure^35,65,66^. It is important to expand our knowledge base to determine if dietary deficiencies and environmental co-exposures (e.g., additional heavy metals, pesticides, wild-fire smoke, fluorine) can combine to make a person more susceptible to developing skeletal deficit.

In summary, in rapidly growing female mice severe, but not marginal, zinc deficiency lowered bone mass and altered cortical and cancellous bone microarchitecture. However, exposure to subchronic, environmentally relevant concentrations of arsenite in drinking water had little effect on bone in zinc-adequate, marginally zinc-deficient, or severely zinc-deficient mice.

## Supplementary Information

Supplementary Information.

## Data Availability

All data generated or analyzed during this study are included in this published article. The datasets will also be made available to anyone upon request.

## Abbreviations

µCT: Microcomputed tomography
As: Arsenite
BMC: Bone mineral content
BMD: Bone mineral density
BMI: Body mass index
DXA: Dual energy X-ray absorptiometry
ICP-MS: Inductively coupled plasma mass spectrometry
ICP-OES: Inductively coupled plasma optical emission spectrometry
MZD: Marginal zinc deficient
SZD: Severe zinc deficient
ZA: Zinc adequate
